# Robust Selection of Cancer Survival Signatures from High-Throughput Genomic Data Using Two-Fold Subsampling

**DOI:** 10.1371/journal.pone.0108818

**Published:** 2014-10-08

**Authors:** Sangkyun Lee, Jörg Rahnenführer, Michel Lang, Katleen De Preter, Pieter Mestdagh, Jan Koster, Rogier Versteeg, Raymond L. Stallings, Luigi Varesio, Shahab Asgharzadeh, Johannes H. Schulte, Kathrin Fielitz, Melanie Schwermer, Katharina Morik, Alexander Schramm

**Affiliations:** 1 Department of Computer Sciences, TU Dortmund University, Dortmund, Germany; 2 Department of Statistics, TU Dortmund University, Dortmund, Germany; 3 Center for Medical Genetics, Ghent University Hospital, Ghent, Belgium; 4 Department of Oncogenomics, Academic Medical Center, Amsterdam, the Netherlands; 5 Cancer Genetics, Royal College of Surgeons, Dublin, Ireland; 6 Laboratory of Molecular Biology, Giannina Gaslini Institute, Genova, Italy; 7 Hematology/Oncology, Children's Hospital Los Angeles, Los Angeles, California, United States of America; 8 Department of Pediatric Oncology and Hematology, University Children's Hospital Essen, Essen, Germany; 9 Centre for Medical Biotechnology, University Duisburg-Essen, Essen, Germany; 10 Translational Neuro-Oncology, West German Cancer Center, University Hospital Essen, University Duisburg-Essen, Essen, Germany; 11 German Cancer Consortium (DKTK), Heidelberg, Germany; 12 German Cancer Research Center (DKFZ), Heidelberg, Germany; Rutgers University, United States of America

## Abstract

Identifying relevant signatures for clinical patient outcome is a fundamental task in high-throughput studies. Signatures, composed of features such as mRNAs, miRNAs, SNPs or other molecular variables, are often non-overlapping, even though they have been identified from similar experiments considering samples with the same type of disease. The lack of a consensus is mostly due to the fact that sample sizes are far smaller than the numbers of candidate features to be considered, and therefore signature selection suffers from large variation. We propose a robust signature selection method that enhances the selection stability of penalized regression algorithms for predicting survival risk. Our method is based on an aggregation of multiple, possibly unstable, signatures obtained with the preconditioned lasso algorithm applied to random (internal) subsamples of a given cohort data, where the aggregated signature is shrunken by a simple thresholding strategy. The resulting method, RS-PL, is conceptually simple and easy to apply, relying on parameters automatically tuned by cross validation. Robust signature selection using RS-PL operates within an (external) subsampling framework to estimate the selection probabilities of features in multiple trials of RS-PL. These probabilities are used for identifying reliable features to be included in a signature. Our method was evaluated on microarray data sets from neuroblastoma, lung adenocarcinoma, and breast cancer patients, extracting robust and relevant signatures for predicting survival risk. Signatures obtained by our method achieved high prediction performance and robustness, consistently over the three data sets. Genes with high selection probability in our robust signatures have been reported as cancer-relevant. The ordering of predictor coefficients associated with signatures was well-preserved across multiple trials of RS-PL, demonstrating the capability of our method for identifying a transferable consensus signature. The software is available as an R package rsig at CRAN (http://cran.r-project.org).

## Introduction

Identification of relevant features from large data sets has been a focus of many research fields for a long time. With the onset of high-throughput genomic profiling technologies, robustness is being perceived as an important factor in feature selection [1], [2]. Generally speaking, a feature is robust if it is chosen by a method invariably of cohort composition, assuming that all samples come from the same population distribution. If an algorithm identifies many of these robust features, then the algorithm can be considered as robust as well. Robustness is a critical factor especially in clinical studies, when the purpose is either to identify the key players in the underlying biological systems, or to develop clinically useful tests.

Unfortunately clinical studies are usually performed without an explicit consideration of robustness in their experimental design. A typical example is to perform feature selection on a single partition of available cohort data, then to determine the success of selection using the rest of data (often called as a test set). When sample sizes are small as in most clinical studies, such practices can lead to identifying diverse signatures from multiple studies that look perfectly fine on their own evaluation but are not successful when they are applied to the data from other studies.

In this paper we propose an algorithm to deal with the aforementioned issues, based on well-studied ideas of subsampling [3] and aggregation [4]. Our framework consists of two subsampling steps: (i) an *outer subsampling* step, which estimates the prediction performance of models and the selection probability of features, and (ii) an *inner subsampling* step, which obtains a robust model by aggregating many, possibly unstable, models, where each model is obtained from a subsample.

In the outer subsampling, we essentially perform bootstrapping [3] to estimate two quantities: the selection probabilities of features and the prediction performance of models composed of robust signatures. The estimation of selection probabilities of features using subsamples has also been used in Davis et al. [1], in the context of choosing the best combination of a feature selection and a separate classification algorithm to maximize both selection frequency of features and classification accuracy. In our method, feature selection and model fitting are performed simultaneously, and it is an intrinsic property that relevant features are to be chosen with high probability. Therefore we use estimated selection probabilities for constructing robust signatures, not for finding the best combination.

The use of aggregation to produce robust signatures as in our inner subsampling step has been used in different contexts. Abeel et al. [5] considered simple and weighted averages of decision vectors from the support vector machines (SVMs) [6] and the recursive feature elimination using SVMs [7], where each decision vector is obtained from a bootstrap sample. In Broom, Do and Subramanian [8], a modified framework has been proposed for leaning structures in Bayesian networks. These works however do not address the problem of identifying robust signatures from censored survival outcome, a typical type of responses in clinical research. Also, methods such as SVMs have no such guarantee that important features will be selected with high probability over different subsamples.

Our robust selection is based on theoretical arguments developed recently for the widely used lasso algorithm [9] and an extension called the preconditioned lasso algorithm [10], that are introduced in the following section.

### Cox Regression with the Lasso Penalty

Let us consider a cohort sample that consists of *n* patients, where each of 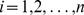 patients is profiled by a *p*-dimensional feature vector x*^i^* and a survival annotation 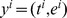: *t^i^* is the length of survival in time and *e^i^* is an indicator for a clinical event such that *e^i^* = 1 if an event has happened, and *e^i^* = 0 otherwise.

In the Cox regression [11], the risk for a patient having an event at time *t* is modeled by a function 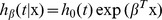, where *h*_0_(*t*) is the baseline hazard function, the exponentiation part describes the effect of covariates, and 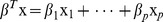. An estimate 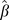 of the coefficient vector *β* is obtained by the maximum likelihood estimation, that is,
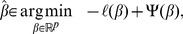
(1)where 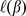 is the partial log-likelihood defined by
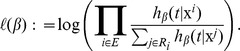



Here *E* is an index set enumerating all events and 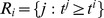 is an index set of patients at risk with respect to the time of an event *i*. The second term in Eq. (1) is a regularizer penalizing the complexity of *β*,
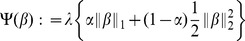
with 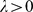 and 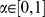. We often call the regularization with *α* = 1 as the lasso or 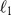, and the one with *α* = 0 as the ridge or 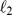 penalty. Lasso selects features by setting the coefficients in *β* to exactly zero for irrelevant features, whereas the ridge does not perform feature selection by itself. For the detailed comparison of the two, we refer to Gui and Li [12]. For 0<*α*<1, the regularizer is called the elastic net [13], which tends to select all correlated covariates together.

### Preconditioned Lasso

The preconditioned lasso algorithm [10] is a two-step procedure designed to address the problems of high bias in lasso estimates when the number of features *p* is very large compared to the number of patients *n*. The two steps are

1. Preconditioning step: 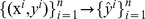.
2. Lasso step: fit a model to 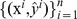.

The first step creates preconditioned outcomes 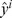 from the given features and survival data. The preconditioning is performed by the supervised principal components method [14], which first ranks features 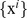 by their individual correlation to survival outcomes 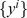, and then find a threshold by cross validation that gives the best prediction performance if the features ranked higher than the threshold are used in regression after being projected onto the first few principal components. The preconditioned outcomes 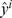 are produced as the result of prediction on each feature vector 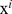 in a training set. Here 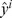 is real-valued, whereas the original outcome 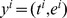 contains a value of survival time and an event indicator.

The second step uses lasso to fit a linear model to the original feature vectors and the preconditioned outcome. Since preconditioned responses 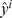 are scalars, we can use the ordinary least squares regression with the lasso penalty,
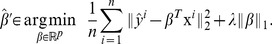
(2)


This problem can be solved efficiently with the least angle regression (LARS) algorithm [15]. After a solution 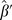 is found, a linear risk prediction 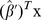 can be computed for each test instance x and compared to their survival risk in forms of the Cox model.

### Consistency and Robust Signature Selection

Suppose that we obtain 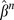 by solving Eq. (1) with *n* examples, where the examples are generated with an unknown population parameter 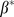 under the Cox model. An important notion in statistics regarding robust feature selection is the *consistency in terms of variable selection*,
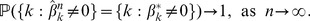
(3)


That is, 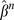 selects the same features to 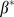 with increasing probability as the number of patients increases. This implies that if *n* is large enough or the convergence in Eq. (3) is fast enough for a fixed *n*, then the feature subsets chosen by several 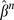 using different samples of size *n* will be the same with high probability, since all of them will be close to the features to be chosen by 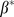. Therefore for robust selection in clinical studies where the number of patients *n* is relatively small and not easy to increase, we prefer to using a method with fast convergence in consistency.

Recently it has been shown that under the *irrepresentable conditions* [16] or equivalently the *neighborhood stability conditions* [17], consistent estimates can be obtained by lasso, although these conditions usually break in real situations. The preconditioned lasso algorithm [10] is an alternative to lasso, producing consistent estimates e.g. when 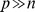. For ordinary least squares with the lasso penalty, it is shown that when the regularization parameter 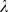 is chosen to be 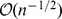, then each active element of 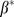 is chosen by 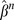 with strictly positive probability [18]. Therefore an intersection of feature sets obtained from bootstrap trials will be nonempty, and be consistent with exponentially increasing probability as *n* grows. However, the arguments are based on strong assumptions that are rather easily violated in practice, and therefore the desired property may not follow. Another modification of lasso has been suggested using random reweighting of the lasso regularizer [19]. This algorithm produces consistent estimates in less restrictive conditions than the previous approach, but requires to specify an extra “weakness” parameter which is not straightforward to determine in its randomized setting.

Our robust selection method is based on the following three critical observations. First, preconditioned lasso has better convergence in consistency than lasso when 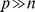 [10]. Second, variation in models can be reduced by model averaging combined with subsampling [4] (inner subsampling step). And third, relevant features are to be selected with positive probability with lasso under certain conditions [18], and therefore will appear more often than irrelevant features in multiple trials with random subsamples (outer subsampling step).

A robust signature is defined as follows: given a random subsample index set 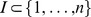 and an estimate 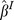 obtained with examples corresponding to *I*, the robustness of a feature indexed by 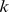 is defined as its probability of being selected amongst all trials with random subsamples,
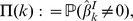
where all parameters, if any, are assumed to be adjusted for each *I*. A *robust signature* is defined as a set of robust features, whose selection probabilities are above a certain threshold 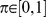, that is,
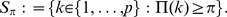



The above two definitions are adapted from Meinshausen and Bühlmann [19]. After evaluating selection probability of features 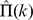 in outer subsampling, we use it to identify an estimated robust signature 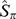,
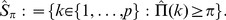
(4)


## Methods

**Figure 1:**
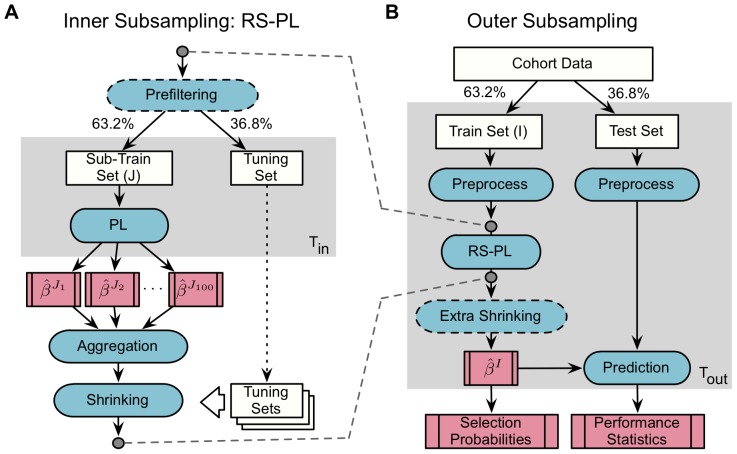
An overview of the suggested framework. Panel A: our core algorithm (abbreviated as RS-PL) performs robust selection with an inner subsampling, using the preconditioned lasso (PL) method inside. Potentially unstable model coefficient vectors 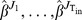 are aggregated and then shrunken to produce a robust model vector 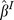. Panel B: an outer subsampling is used to evaluate the prediction performance of RS-PL and to estimate selection probabilities of features. The ratios (63.2%∶36.8%) are chosen to resemble the effective sampling ratios in bootstrapping.

The workflow of our newly developed method is sketched in Figure 1. The left panel (A) shows RS-PL, our Robust Selection procedure with the Preconditioned Lasso algorithm, which produces a coefficient vector 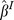 for each random train index set *I*. In the right panel (B), we estimate the selection probability of each feature chosen by the RS-PL algorithm for each random train set *I*, testing the performance of predictors as well.

Our method RS-PL is designed to enhance the robustness of lasso-based signature selection methods, in particular the preconditioned lasso (PL). PL and RS-PL perform both signature selection and estimation of a prediction function at the same time in a tightly coupled manner. Therefore, improving robustness in signature selection tends to improve prediction performance. More specifically, predictors of RS-PL are based on an ensemble of linear models of chosen features, and therefore robustness in signature selection is directly connected to the stability of ensemble models and their prediction outcome.

### Robust Selection with Preconditioned Lasso (RS-PL)

Our suggested algorithm RS-PL in Figure 1 (A) corresponds to an inner subsampling step in the entire framework, where a train index set *I* is split into a sub-train set *J* (63.2%) and a tuning set (the rest). These ratios are chosen to resemble the effective number of samples in bootstrapping [3]. In comparison to other subsampling strategies such as *k*-fold cross validation, this particular way of subsampling is known to provide the best estimation when noise in data is moderate [20].

#### Prefiltering

In RS-PL, we first remove uninformative features from each train set (I) whose standard deviation values are below a predefined percentile of the standard deviation values of all features. This filtering is optional but facilitates feature selection. In particular, a desirable number of candidate features *p* can be determined using Lemma 6.7 [21], which states that the number of features 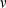 to be chosen with statistical consistency with the lasso and the preconditioned lasso is bounded by 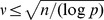 for a sample of size *n*. In other words, *p* should be no larger than 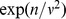. For instance, *p* could be up to a few thousands when *n* = 176 and 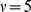. In our experiments we expected that 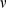 would be 5∼10 and reduced the number of candidate features as suggested by the lemma using prefiltering.

#### Preconditioned Lasso

At the core of RS-PL, we use the preconditioned lasso algorithm (abbreviated as PL) discussed above, because of its superior characteristics for the cases with 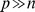. PL inside of RS-PL can be replaced by other algorithms as long as they produce coefficient vectors for linear models, such as the Cox regression with the lasso penalty.

#### Aggregation and Shrinking of Signatures

For each sub-train set 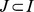, we obtain an estimate coefficient vector 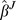 as a result of solving the second step of preconditioned lasso in Eq. (2). For T_in_ = 100 random sub-train sets, say 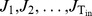, we obtain estimated coefficient vectors 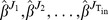 respectively. Since the coefficient vectors are from linear models, we can aggregate them by a simple averaging, that is,
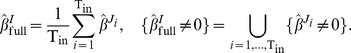



Here the aggregated coefficient vector 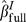 is denoted with the letter *I*, since it is produced for each train set *I* in effect.

The number of features to be selected by the aggregated vector 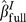 tends to be quite large, since the set of nonzero components in 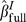 is the same as the union of signatures obtained with 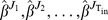, as indicated above. Therefore we “shrink” the coefficients in 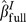 using a simple thresholding strategy: for threshold values 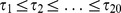 where 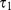 and 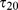 are the smallest and the largest magnitude of components in 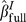, we find a threshold 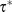 such that the shrunken signature 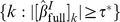 and its corresponding coefficients produce the best prediction results over tuning sets, where tuning sets come from the inner subsampling in Figure 1 (A). We denote the aggregated and shrunken robust coefficient vector, the final outcome of RS-PL, as 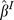, constructed as follows:
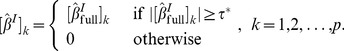
(5)


### Estimation of Selection Probability, Prediction Performance, and Robustness

The algorithm in Figure 1 (B) corresponds to an outer subsampling step, where the entire cohort data with *n* patients are split into a train set *I* (63.2%) and a test set (the rest), randomly for T_out_ = 100 times.

#### Preprocessing

There are two separate preprocessing steps for each train set (*I*) and each test set. This separation is quite important for accurate estimation of prediction performance. For example, when we apply summarization and normalization algorithms such as the robust multi-array analysis (RMA) [22] to microarray data, we need to apply RMA separately on a train set and a test set, since otherwise RMA will use information from a test set to preprocess a train set, and vice versa, and therefore such practice can yield overly optimistic prediction accuracy estimations on the test set.

Alternatively, the frozen RMA (fRMA) algorithm [23] can be applied independently to individual microarrays, using global reference microarrays for normalization. Due to independence, fRMA needs to be applied only once for all arrays regardless of train/test splits.

#### Prediction of Risk

For prediction, a robust and shrunken coefficient estimate 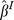 in Eq. (5) obtained by RS-PL is used to compare the risk of patients having an event at time *t*, in terms of the Cox proportional hazard model [11]. In this model, the log hazard ratio comparing the risk of two patients (with profiles 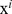 and 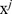) becomes
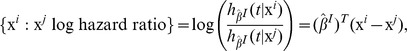
from the definition of the hazard (risk) function 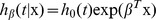. The hazard ratio provides a statistic for testing differences in survival patterns. It is worthwhile to note that the baseline hazard *h*_0_(*t*) is cancelled out and does not play any role in the above expression, making comparison of risk as simple as comparing the values of linear predictors 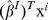 and 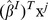. This enables us to use a rank correlation between linear predictors and survival times to assess prediction performance, as we discuss in the next section.

On the other hand, the baseline hazard *h*_0_(*t*) can be estimated in order to produce survival probabilities for individual patients. An estimate of *h*_0_(*t*) is suggested by Cox and Oakes [24],
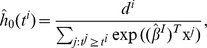
where 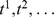 are the distinct event times and *d^i^* is the number of events at *t^i^*. Then the survival function (the probability to survive at least to time *t*) for a patient x can be computed by,
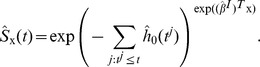



#### Measures for Prediction Performance

To measure prediction performance, we use the *concordance index* [25], which is the fraction of all comparable patient pairs whose outcomes are concordant to predictions. A pair of patients is considered to be usable except for the cases where both patients have events at the same time, or one has shorter censored survival time than the other who has an event. To explain formally, suppose that a prediction 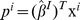 is available for each patient 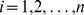 whose survival time is given by 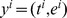 with an event indicator 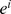. Consider the following order indicator functions [26] for 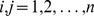,
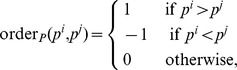


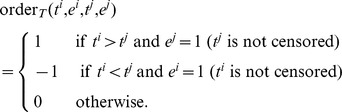



Then the product of the two order functions measures if the order of a pair of predictions is concordant (product  = 1), disconcordant (−1), or not comparable (0) to the order of the corresponding survival time pair. The concordance index is defined as the fraction of concordant pairs among all comparable pairs,
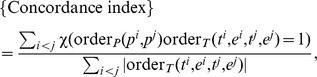
which has a value between 0 and 1. Here 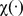 is an indicator function returning 1 if the argument is true, and 0 otherwise. Note that the numerator above counts the number of all concordant pairs, where the denominator counts the number of all comparable pairs (concordant or disconcordant). This measure can be described as a generalized AUC (area under the ROC curve) value, where values>0.5 imply positive correlation and values <0.5 imply negative correlation. For binary valued predictions, the concordance index becomes identical to the AUC.

#### Measures for Robustness

In order to measure robustness of signature selection, we use the *Jaccard index* and the *rank-penalized Kuncheva index*.

The *Jaccard index* measures the robustness of signatures of possibly different sizes, and it is defined as an average size of overlap between feature subsets relative to the size of their union [2]. Denoting the set of features chosen with 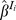 by 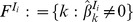, it is defined as:
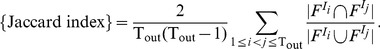
(6)


The Jaccard index ranges from 0 to 1, and larger values indicate larger relative overlap.

When the sizes of signatures can be controlled, more precise measures of robustness are available, namely the Kuncheva index [27] and the Canberra distance [28], instead of the Jaccard index which can result in a biased evaluation of robustness. Specifically, the Kuncheva index provides an unbiased estimate of average overlap between signatures, and the Canberra distance measures how well the order of contribution of features is preserved between signatures on average. Compared to the Jaccard index, these two measures require signatures to be of the same size for comparison. The fraction between the Kuncheva index and the Canberra distance, denoted as the *rank-penalized Kuncheva index*, is computed as a summary of the two measures of robustness. Denoting the 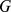 features chosen from 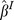 in an extra shrinkage by 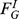, and the rank in magnitude of the 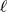
th feature in 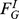 by 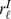, the rank-penalized Kuncheva index is expressed as follows (*p* is the total number of candidate features),
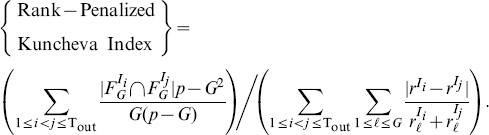
(7)


The values of this index range from 0 (zero overlap, i.e., feature ranks not preserved) to ∞ (perfect overlap, i.e., perfect preservation of feature ranks).

#### Extra Shrinkage of Models

The number of features in a signature described by 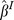 varies depending on data and methods, but it is typically larger than 50. When smaller signatures are preferred for an in-depth investigation of features, signatures described by 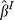 can be shrunken further by choosing the top *G* features according to the magnitude of their coefficient in 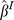.

This is subsequently used for an evaluation of our method to compare robustness and prediction performance of signatures consisting of small numbers of features.

#### Selection Probabilities of Features and Robust Signatures

The selection probability of a feature, indexed by *k*, is estimated by its appearance frequency among the T_out_ outer subsampling trials, that is,
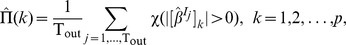
where 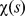 is an indicator function which equals to 1 if the statement *s* is true, or 0 otherwise. Given these probabilities and a baseline selection probability *π*, we construct a robust signature according to Eq. (4).

### List of Algorithms for Comparison

Our suggested algorithm **RS-PL**, where the prefix “RS” stands for “robust selection”, is compared to the following algorithms. **RS-L** is the same as RS-PL, except that PL inside of RS-PL is replaced with the Cox regression with the lasso penalty. In the following, the entire RS-PL in Figure 1 (A) is replaced with the described algorithms, that do not make use of our RS framework: **PL** is the preconditioned lasso algorithm. **L** is the Cox regression with the lasso penalty. **Dev** is a simple method that selects the top 100 features with the largest standard deviation across microarrays. A ridge Cox regression is then performed, using only these features. This type of methods is known to be stable [29]. **Cor** is another univariate method, choosing the top 100 features with the highest ranks in terms of their individual correlation to survival annotation (measured by the concordance index). A ridge Cox regression is performed on the selected features afterwards. **Cli** is a Cox regression without penalty using only clinical covariates. The BatchExperiments package [30] for R was used for parallel computation of algorithms.

## Results

### Data Preparation

Three data sets were analyzed containing mRNA expression profiles from a total of 742 cancer patients that were acquired by using Affymetrix microarray technology. Data were obtained for three different entities, neuroblastoma, lung adenocarcinoma, and breast cancer, as summarized in Table 1. CEL files were downloaded from the Gene Expression Omnibus or the R2 platform (http://r2.amc.nl). For preprocessing, the frozen RMA algorithm [23] was applied to individual CEL files to create probeset level summaries. Only microarrays with the median GNUSE [31] values ≤1 (for quality control) and with appropriate clinical information (overall survival) were included in this study. The characteristics of three data sets before and after preprocessing are summarized in Tables 2, 3, and 4 (see Figure S1 for the corresponding Kaplan-Meier plots).

**Table 1:** Three data sets for evaluation.

Data Set	Source	Platform	*n*
Neuroblastoma	GSE21713, GSE32664, and R2*	Human Exon ST v1.0	176
Adenocarcinoma	GSE31210	HG-U133 Plus 2	204
Breast Cancer	GSE1456, GSE7390, GSE11121	HG-U133A	362

*R2: microarray analysis and visualization platform (http://r2.amc.nl).

**Table 2:** Characteristic of patients before/after GNUSE filtering (neuroblastoma).

Categories	Groups	Before (*n* = 295)	After (*n* = 176)
Age (yrs)	≤1:>1:NA	98∶192∶5	56∶120∶0
INSS stages	1∶2∶3∶4∶4s:NA	58∶40∶47∶130∶17∶3	23∶26∶31∶90∶6∶0
MYCN status	Single:Amplified:NA	232∶57∶6	133∶41∶2
Survival time (yrs)	≤5 (with event):≤5 (no event):> 5:NA	73∶101∶114∶7	52∶65∶59∶0

Microarrays with median GNUSE scores>1.0 and with no overall survival time annotation were discarded (NA: not available).

**Table 3:** Characteristic of patients before/after GNUSE filtering (adenocarcinoma).

Categories	Groups	Before (*n* = 246)	After (*n* = 204)
Age (yrs)	Min:Med:Max:NA	30∶61∶89∶66	30∶61∶76∶37
Smoking status	Ever:Never:NA	123∶123∶0	99∶105∶0
MYC status	High:Low:NA	17∶207∶22	16∶187∶1
Survival time (yrs)	≤5 (with event):≤5 (no event):> 5:NA	32∶93∶101∶20	27∶81∶96∶0

Microarrays with median GNUSE scores>1.0 and with no overall survival time annotation were discarded (NA: not available).

**Table 4:** Characteristic of patients before/after GNUSE filtering (breast cancer).

Categories	Groups	Before (*n* = 514)	After (*n* = 362)
Age (yrs)	Min:Med:Max:NA	24∶51.53∶89.65∶159	24∶55.45∶83.17∶150
Grade	1∶2∶3:NA	81∶253∶166∶14	60∶180∶112∶10
Survival time (yrs)	≤5 (with event):≤5 (no event):> 5:NA	74∶15∶425∶0	54∶5∶303∶0

Microarrays with median GNUSE scores>1.0 and with no overall survival time annotation were discarded (NA: not available).

The features obtained from preprocessing are denoted by *probesets*, which correspond to (parts of) exons or genes depending on microarray platforms. The total numbers of probesets (features) differ depending on microarray platforms: HG-U133A Plus 2.0 platform contains 54675 probesets (HG-U133A contains about 10000 less probesets), and Human Exon ST v1.0 platform contains 1432143 probesets, according to the NetAffx probeset annotation v33.1 from Affymetrix. Each probeset has a summarized expression values of corresponding *probes* in the original CEL data, where 9∼11 (HG-U133A) or 1∼4 (Human Exon ST v1.0) probes constitute a probeset. For the neuroblastoma data set (Human Exon ST v1.0), we focused on the core level probesets as features corresponding to exons that fulfilled three criteria: unique hybridization, unique localization on one of the human chromosomes, and the presence of valid gene assignments. Using the NetAffx probeset annotation, this resulted in 228476 features. When prefiltering was applied, the probesets with standard deviation less than the 99th percentile of the standard deviation of all features were discarded for each random train set *I*, resulting in 2285 features. For adenocarcinoma (HG-U133 Plus 2) and breast cancer (HG-U133A) data sets, we focused on the grade-A probesets as features corresponding to genes with unique hybridization and unique localization. Using the NetAffx annotation, this resulted in 28476 (adenocarcinoma) and 20492 (breast cancer) features, respectively. When prefiltering was applied, the probesets with standard deviation less than the 90th percentile of the standard deviation of all features were discarded for each random train set *I*, resulting in 2848 (adenocarcinoma) and 2050 (breast cancer) features.

Clinical covariates were used only for the method Cli, including the following attributes: age at diagnosis, MYCN status and INSS stage for neuroblastoma; age, smoking status, gender, stage, and MYC status for lung adenocarcinoma; age, stage, size of tumor, and grade for breast cancer.

### Robust Signatures

The algorithms RS-PL, RS-L, PL, L, Dev, Cor and Cli were tested within our evaluation framework (Figure 1: B), using the same random splits of data across different methods for fair comparison (see Table S1 for survival time distribution of train and test sets). This resulted in a sequence of coefficient vectors 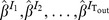 as an output of each method. These were used to estimate the selection probability of each feature, 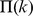. For the neuroblastoma data set, the baseline probability 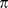 was set to the estimated selection probability of the MYCN amplification status covariate (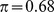). For the other two data sets, an arbitrary value (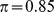) was defined and robust signatures were obtained.

#### Qualitative Validation of Robust Signatures

Tables 5, 6, and 7 show the features included in robust signatures produced by RS-PL, for neuroblastoma, lung adenocarcinoma, and breast cancer, respectively (see Tables S2, S3, and S4 for the corresponding lists of chosen features and their selection probability). In each table, selection frequencies of features are shown in the second column. As for neuroblastoma, data were available with exon level resolution, so selection frequency values were averaged over multiple exons if more than one exon was stably identified for a gene. Selection of multiple exons for a single gene (Table 5) could imply differential exon usage, which has already been proven for NTRK1 expression in neuroblastoma: NTRK1 isoforms have been reported to be associated with different patient outcome [32]. TMEFF2 is a PDGF-AA binding protein associated with gene silencing [33], while PDGF-AA is known to be functional in neuroblastoma cell growth [34]. SCN7A and CHD5 have all been linked causally to neuroblastoma biology and prognosis [35], [36]. The other genes were supported by various literature (Table 5). Several genes identified by RS-PL were also supported by literature in lung cancer (Table 6: LY75-CD302, PLAUR, FAM184A, BUB1B, MBM4, CCNB2, SUSD2, HJURP, and CYP4B1) and breast cancer (Table 7: MELK, CDC20, FRZB, UBE2C, LAMA2, SCUBE2, MMP1, FBLN1, PDGFD, RRM2, and SPARCL1). Taken together, these findings demonstrate that RS-PL is capable of identifying biologically meaningful signatures and potentially important biomarkers.

**Table 5:** A robust signature obtained with RS-PL from the neuroblastoma data set (*π* = 0.68).

Gene	Mean Frequency	# Selected Exons	Relevance	Rank in Dev	Rank in Cor
NTRK1	0.90	3	Neuroblastoma [32]	-, -, 112	110, 145, 177
TMEFF2	0.87	1	Neuroblastoma [33], [34]	-	50
FAM70A	0.85	1	Neuroblastoma [37]	-	217
SCN7A	0.83	2	Neuroblastoma [35]	-, -	48, 234
AKR1C2	0.82	1	Neuroblastoma [38]	-	69
SLC18A2	0.82	1	Brain diseases	-	632
CHD5	0.81	4	Neuroblastoma [36]	-, -, -, -	12, 30, 76, 87
RGS9	0.81	2	Brain diseases	-, -	-, 225
ANKFN1	0.80	1	Brain development disorders	-	660
LRGUK	0.78	1	Neuroblastoma [39]	-	819
POF1B	0.76	1	Brain development disorders [40]	-	307
ADRB2	0.75	1	Neuroblastoma [41]	-	-
AMIGO2	0.74	2	Neuroblastoma [37]	-, -	-, 1236
PMP22	0.74	1	Neuroblastoma [42]	69	54
ARHGAP36	0.71	1	Neuroblastoma [43]	27	-
HS3ST5	0.70	1	Brain diseases [44]	-	-
MDGA1	0.70	1	Brain diseases	-	74
PGM2L1	0.69	1	Neuroblastoma [45]	-	837
EPB41L4A	0.68	1	Other cancers	-	-
SOX6	0.68	1	Neuroblastoma [46]	-	437

The second column shows the mean values of selection probabilities of exon features. Multiple selection of exons from a single gene suggests differential expression, while the others indicate possible mutations. The relevance of features without references were extracted from the GeneCards (http://www.genecards.org). The corresponding ranks of chosen features (probesets) in Dev and Cor methods are shown in the last two columns (‘-’ if not chosen).

**Table 6:** A robust signature obtained with RS-PL from the lung adenocarcinoma data set (*π* = 0.85).

Gene	Frequency	Relevance	Rank in Dev	Rank in Cor
CD302/LY75-CD302	1.0	Lung cancer [47]	-	1078
SCN4B	1.0	Lung cancer [48]	-	-
HLF	0.98	Other cancers	-	-
FBXO32	0.97	Other cancers [49]	-	539
PLAUR	0.97	Lung cancer [50]	-	180
COL11A1	0.96	Other cancers	19	-
FAM184A	0.94	Lung adenocarcinoma [51]	-	-
BUB1B	0.93	Lung cancer [52]	-	1018
MCM4	0.93	Lung cancer [53]	-	41
CCNB2	0.92	Lung adenocarcinoma [54]	-	235
SUSD2	0.92	Lung cancer [55]	56	-
GPR116	0.91	Lung function [56]	-	-
HJURP	0.90	Lung cancer [57]	-	-
CYP4B1	0.89	Lung cancer* [58]	21	1038
GFRA1	0.89	Other cancers	-	1670
GPR84	0.88	-	-	500
LOC100499467	0.88	-	-	348
SLC12A8	0.88	-	-	-
DLGAP5	0.86	Other cancers	-	-

*It was reported to the contrary that CYP4B1 was normally expressed in lung cancer patients [58]. If the relevance of features was unclear or unknown, it was marked with hyphens.

**Table 7:** A robust signature obtained with RS-PL from the breast cancer data set (*π* = 0.0.85).

Gene	Frequency	Relevance	Rank in Dev	Rank in Cor
MELK	0.96	Breast cancer [59]	-	58
ZCCHC24 (212419_at)	0.96	Breast Cancer [60]	-	17
COL14A1	0.93	Other cancers	-	73
ZCCHC24 (212413_at)	0.92	Breast Cancer [60]	-	203
CDC20	0.92	Breast cancer [61]	-	196
FRZB	0.91	Breast cancer [62]	-	1
IGJ	0.91	-	53	32
UBE2C	0.91	Breast cancer [63]	-	690
LAMA2	0.90	Breast cancer [64]	-	8
SCUBE2	0.90	Breast cancer [65]	28	-
MMP1	0.89	Breast cancer [66]	27	-
FBLN1	0.88	Breast cancer [64], [67]	-	82
IGH@/IGHA1/IGHA2	0.88	-	47	10
PDGFD	0.87	Breast cancer [68]	-	106
RRM2	0.87	Breast cancer [69]	-	213
SPARCL1	0.87	Breast cancer [70]	-	48

For ZCCHC24, two transcripts (with probeset IDs 212419_at and 212413_at) were chosen.

### Overall Prediction Performance and Robustness

**Figure 2:**
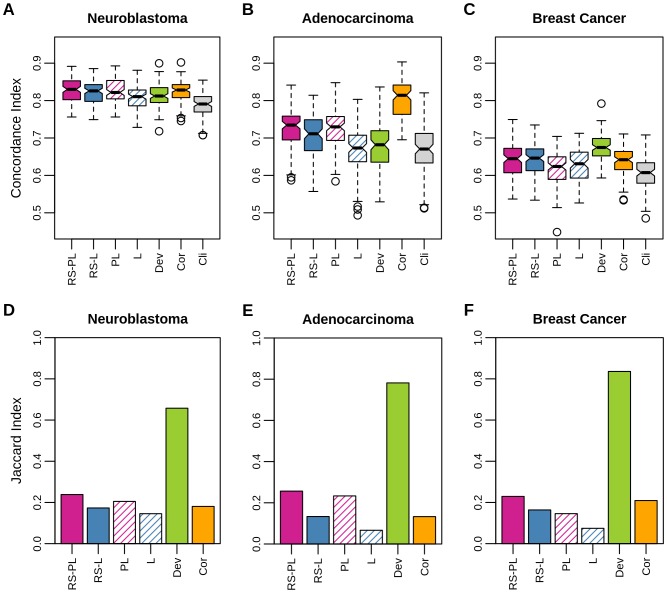
Overall prediction performance and robustness. Prediction performance in the concordance index (panels A, B, and C) and robustness in the Jaccard index (panels D, E, and F) are shown respectively for neuroblastoma (A/D), lung adenocarcinoma (B/E), and breast cancer (C/F) data sets. Bands inside of boxes represent median values (A–C). In prediction, the proposed method RS-PL was on a par with Cor but better than the rest (one-sided paired Welch's t-test, *p*<0.001) for neuroblastoma, and the second best for adenocarcinoma and breast cancer. Robustness of RS-PL was the highest except for Dev.

Figure 2 shows the prediction performance (panels A–C) and the robustness (panels D–F) of methods over T_out_ outer subsampling trials for the three data sets used here (in columns), in terms of the concordance index for predicting survival risk of test patients and of the Jaccard index (Eq. (6)), respectively. In this figure the numbers of selected features were not necessarily the same, where the numbers would have affected prediction performance. As a result, the comparison of prediction performance among different methods may not be completely fair in Figure 2. Still, it shows the maximal prediction performance when signatures can be flexible in their size.

#### Prediction Performance

The prediction performance of PL and L was improved by the use of our proposed robust selection (RS) framework (Figure 2: A–C). The improvements were significant in the following cases: RS-PL> PL for breast cancer (*p*<10^−9^); RS-L> L for neuroblastoma (*p*<10^−16^), adenocarcinoma (*p*<0.001), and breast cancer (*p*<10^−6^). These results were remarkable since the intent of our RS framework was to improve robustness, but not necessarily to improve prediction performance. On the other hand, these results also revealed the susceptibility of PL and L to overfitting when sample size was smaller than the number of features.

Comparing the prediction performance of our method RS-PL to the others, RS-PL was the best performing, or the second best but consistently well performing across different data sets. For neuroblastoma, RS-PL performed better in terms of prediction performance than RS-L (*p*<0.1) and significantly better than PL (*p*<0.001), L, Dev, and Cli (*p*<10^−9^). There was no significant difference between RS-PL and Cor. The prediction performance of RS-PL was the second best in cases of adenocarcinoma and breast cancer, following Cor and Dev, respectively. However, the prediction performance of Cor and Dev were inconsistent considering their ranks of performance over the three data sets: Cor was ranked at 2nd (neuroblastoma), 1st (adenocarcinoma), and 4th (breast cancer); Dev was ranked at 5th (neuroblastoma/adenocarcinoma) and 1st (breast cancer), considering their median prediction performance. Notably, the performance of Cli was the worst in every case, supporting the use of high-throughput genomic data for risk prediction. (For survival probability predictions of individual patients, see Tables S5, S6, and S7 for neuroblastoma, lung adenocarcinoma, and breast cancer, respectively.)

#### Robustness

The robustness of PL and L was improved by the RS framework (Figure 2: D–F), achieving our main objective: improvements were about 10∼57% (RS-PL vs. PL) and 20∼120% (RS-L vs. L), depending on data sets for which the algorithms were tried.

Overall, RS-PL was the most robust except for Dev. In fact, the robustness indices of both Dev and Cor were consistently high for all the three data sets tried. However, the Jaccard index used here for measuring robustness has several limitations, despite its capability of comparing feature subsets of different sizes: first, the Jaccard index is biased since it does not take into account of a correction for chance selection; second, it completely ignores how well the ranks of features are preserved amongst different selections. Therefore, an alternative measure for robustness was considered subsequently for better evaluation.

#### Prediction Performance vs. Robustness

**Figure 3:**
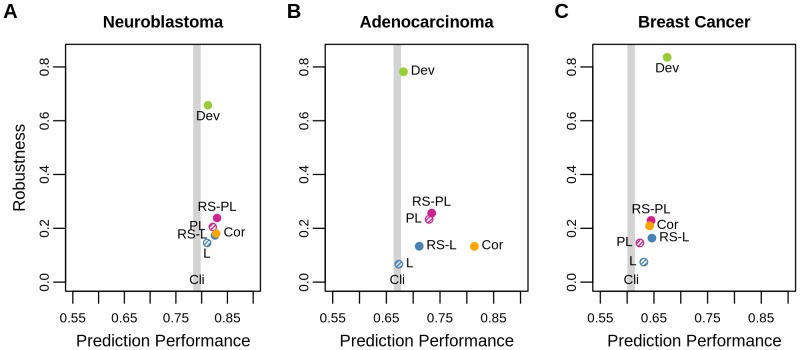
Prediction performance vs. robustness. Prediction performance in the median concordance index (x-axis) and robustness in the Jaccard index (y-axis) are shown respectively for neuroblastoma (panel A), adenocarcinoma (B), and breast cancer (C) data sets. Since no variable selection is performed for Cli, only its prediction performance is shown as vertical lines.

Figure 3 positions the seven algorithms in terms of the two performance criteria, prediction (median concordance index) and robustness (Jaccard index), providing a clear view for comparison. Neuroblastoma: RS-PL was the best performing considering the two measures. Dev was more robust than RS-PL, but its prediction performance was not competent at all. Adenocarcinoma: RS-PL was still the best except for the two extreme cases, Dev (best robustness/poor prediction performance) and Cor (best prediction performance/poor robustness). Breast cancer: Dev was the best performing method in both criteria, being followed by RS-PL and RS-L.

Overall, RS-PL outperformed the other multivariate selection methods (RS-L, PL, and L). The univariate selection methods (Cor and Dev) were better than RS-PL in certain cases, but their performance was inconsistent when they were considered on multiple data sets.

### In-Depth Performance Analysis with an Extra Shrinkage

For an accurate comparison of signatures, it is necessary to produce signatures of the same size from all methods. For this purpose, we applied an extra shrinkage to all selection algorithms by choosing the *G* features with the largest magnitude coefficients in 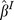, so that the same number of features was selected for each random train index set *I*. This allows for using the rank-penalized Kunchvea index (Eq. (7)) instead of the Jaccard index for a more precise estimation of robustness.

#### Prediction Performance of Small Signatures

Although the use of the extra shrinkage here was for making the rank-penalized Kuncheva index available, it also provided a new perspective on the prediction performance of models consisting of small signatures.

**Figure 4:**
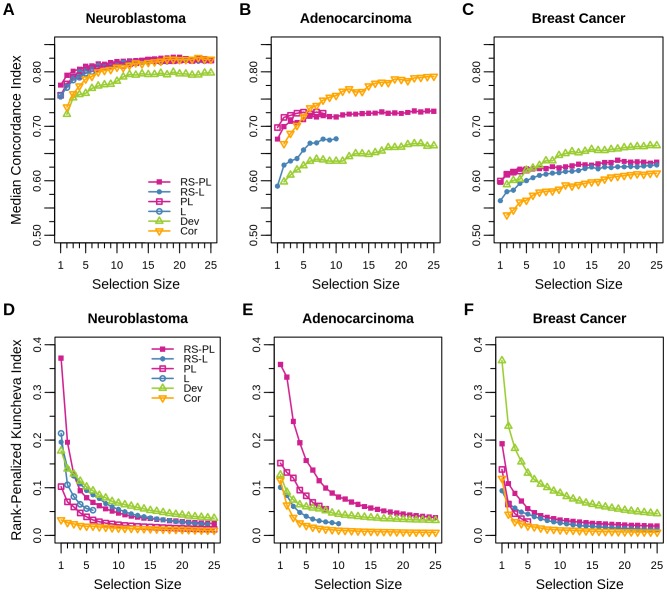
Prediction performance and robustness with an extra shrinkage. Prediction performance in terms of the median concordance index (panels A, B, and C) and robustness in the rank-penalized Kuncheva index (panels D, E, and F) are shown respectively for neuroblastoma (A/D), lung adenocarcinoma (B/E), and breast cancer (C/F) data sets. Signatures of different sizes (denoted by selection sizes) were created in the extra shrinkage step, by choosing the features in 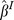 with the largest magnitude coefficients. Values are not plotted for the cases where any of T_out_ trials has chosen less than a specified selection size before the extra shrinkage.

In Figure 4: A–C, the prediction performance values in terms of the median concordance index for signatures of varying sizes *G* (denoted by selection sizes) from 1 to 25 are shown (Cli is not included since it does not perform any variable selection). Comparing to the median prediction performance without extra shrinkage (Figure 2: A–C), the results of RS-PL showed that similar prediction performance values were already achieved by using only ∼20 features in case of neuroblastoma, whereas>25 features were expected to achieve similar prediction performance for adenocarcinoma and breast cancer data sets.

#### Robustness of Small Signatures

Figure 4: D–F reports the robustness of algorithms in terms of the rank-penalized Kuncheva index, for small signatures of varying sizes up to 25.

In these results, the robustness of Cor was consistently the worst in the three data sets, although it showed competent robustness in terms of the Jaccard index previously (Figure 2: D–F). The reason was that even though similar features were chosen by Cor in multiple trials, the ranks of features were not preserved. Dev showed the same issue in case of the adenocarcinoma data set. These results indicated that despite their high prediction performance in certain cases, predictors obtained by Cor and Dev from one data set may not transferable to other data sets: they may produce poor prediction outcome or different prioritization of features if applied to other data sets.

Comparing to the previous evaluation of robustness (Figure 2: D–F), RS-PL was still behind of Dev for the cases of neuroblastoma and breast cancer, but it became the most robust for the adenocarcinoma data set: the reason was that the feature ranks were well preserved by RS-PL, but not by Dev. Overall, RS-PL and Dev performed consistently well in terms of robustness compared to the other methods, but the prediction performance of Dev was not competent to RS-PL in two cases (neuroblastoma and adenocarcinoma).

## Conclusion

Our robust selection (RS) framework successfully improved the robustness of the popular multivariate signature selection methods, the lasso (L) and the preconditioned lasso (PL) algorithms, for predicting survival risk: this was the primary goal of this paper. The goal was achieved by using an ensemble average of potentially unstable models obtained with subsamples, where the averaged model typically had a reduced variance compared to the original models. Large signatures were obtained by such averaged models, but shrinking enabled the identification of compact signatures with negligible effects to prediction performance and robustness (data not shown).

Prediction performance of L and PL was also improved by our RS framework, sometimes with high significance, which was an advantage although it was not necessarily intended. The suggested algorithm, RS-PL, was the best performing in prediction and robustness amongst the multivariate signature selection methods (RS-PL, RS-L, PL, and L). Signatures identified by RS-PL were well supported by literature, constituting a qualitative validation.

For the comparison of RS-PL (multivariate selection) to Cor and Dev (popular univariate selection methods in clinical studies), mixed results were obtained on different data sets. The best performing methods were: RS-PL for neuroblastoma (in terms of both prediction and robustness); Cor (in prediction) and RS-PL (in robustness evaluated with the rank-penalized Kuncheva index) for lung adenocarcinoma; Dev (in both measures) for breast cancer. However, as shown in our results, the performance of Cor and Dev was inconsistent compared to that of RS-PL across multiple data sets. To the contrary, the performance of RS-PL, which was the best or the second best to Cor/Dev, was consistent, indicating that RS-PL can compensate the inconsistency of these univariate selection methods (in practice, trying all the three methods (RS-PL, Cor, and Dev) will be recommended for a given data). Arguments for this aspect leave room for further investigation however, since our experiments were not explicitly designed for validating this aspect (especially the selection size of Cor and Dev were fixed to 100 in our results, which can be adjusted by cross validation or false-discovery-rate control).

Since our method is based on generalized linear models that are capable of handling both continuous and discrete features, it can be applied to the next generation sequencing data and a mixture of expression and sequencing data in principle. However, it is worthwhile to note that a large number of candidate features makes it challenging to discover signatures with statistical power. For example, according to Meinshausen and Bühlmann [19], only the top few features will be statistically meaningful in our setting. The best option will be increasing the number of patients, but it is typically not plausible in clinical studies. Therefore, it is still an open question how to properly handle a large number of features given a small number of patients.

## Supporting Information

Figure S1 **Kaplan-Meier plots of survival times in neuroblastoma, lung adenocarcinoma, and breast cancer patients before and after preprocessing.**
(TIF)

Table S1 **Survival time distribution in train and test sets.** The numbers of patients for groups (≤5 yrs with event, ≤5 yrs without event, and>5 yrs) are averaged over 100 pairs of (train, test) sets.
(DOC)

Table S2 **Lists of chosen features and their selection probability (neuroblastoma).**
(XLS)

Table S3 **Lists of chosen features and their selection probability (lung adenocarcinoma).**
(XLS)

Table S4 **Lists of chosen features and their selection probability (breast cancer).**
(XLS)

Table S5 **Predictions of survival probability (neuroblastoma).**
(XLS)

Table S6 **Predictions of survival probability (lung adenocarcinoma).**
(XLS)

Table S7 **Predictions of survival probability (breast cancer).**
(XLS)
