# Supplementary figures and images for: Robust Selection of Cancer Survival Signatures from High-Throughput Genomic Data Using Two-Fold Subsampling
Source: PLoS One. 2014 Oct 8;9(10):e108818. doi: 10.1371/journal.pone.0108818 (PMC4190101; doi:10.1371/journal.pone.0108818)

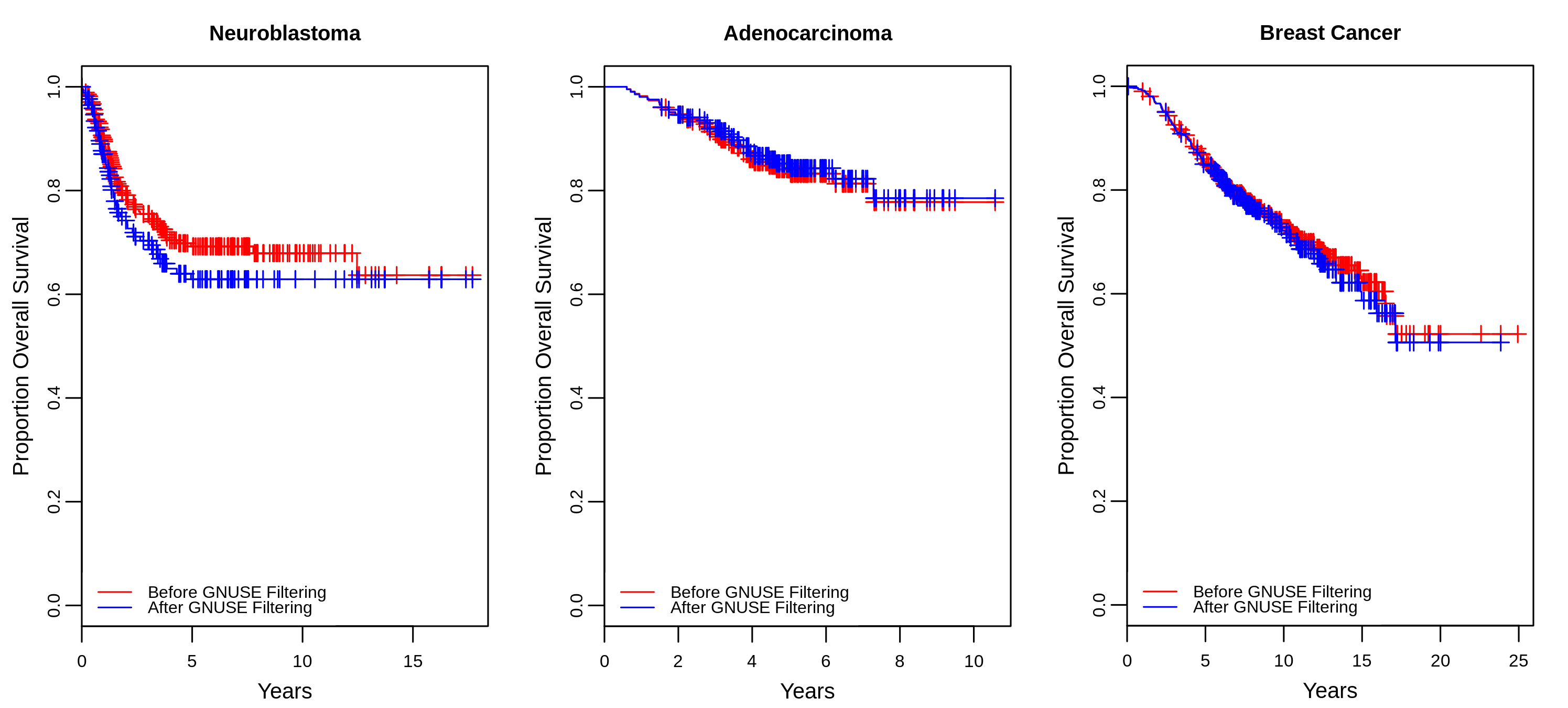

Supplement: Figure S1 — Kaplan-Meier plots of survival times in neuroblastoma, lung adenocarcinoma, and breast cancer patients before and after preprocessing. (TIF) [file pone.0108818.s001.tif]
