# Supplementary material for: Robust Selection of Cancer Survival Signatures from High-Throughput Genomic Data Using Two-Fold Subsampling
Source: PLoS One. 2014 Oct 8;9(10):e108818. doi: 10.1371/journal.pone.0108818 (PMC4190101; doi:10.1371/journal.pone.0108818)
Supplement: Table S1 — Survival time distribution in train and test sets. The numbers of patients for groups (≤5 yrs with event, ≤5 yrs without event, and>5 yrs) are averaged over 100 pairs of (train, test) sets. (DOC) [file pone.0108818.s002.doc]

| Data Set | Train | | | Test | | |
| --- | --- | --- | --- | --- | --- | --- |
|  | <= 5yrs (with event) | <= 5yrs (no event) | > 5yrs | <= 5yrs (with event) | <= 5 yrs (no event) | > 5yrs |
| Neuroblastoma | 32.0 | 40.8 | 37.2 | 20.0 | 24.2 | 21.8 |
| Adenocarcinoma | 16.2 | 51.2 | 59.7 | 10.8 | 29.8 | 36.4 |
| Breast cancer | 33.7 | 3.1 | 191.2 | 20.3 | 1.9 | 111.8 |
